# Purpose Formulation, Coalition Building, and Evidence Use in Public–Academic Partnerships: Web-Based Survey Study

**DOI:** 10.2196/29288

**Published:** 2022-01-05

**Authors:** Christina D Kang-Yi, Amy Page

**Affiliations:** 1 Department of Psychiatry, Perelman School of Medicine, University of Pennsylvania Philadelphia, PA United States; 2 Leonard Davis Institute of Health Economics, University of Pennsylvania Philadelphia, PA United States; 3 Graduate School of Social Service, Fordham University New York City, NY United States

**Keywords:** use of research evidence, public care policy, public–academic partnership, partnership purpose formulation, partnership coalition building, youth mental health and well-being

## Abstract

**Background:**

Partnerships between academic institutions and public care agencies (public–academic partnerships [PAPs]) can promote effective policy making and care delivery. Public care agencies are often engaged in PAPs for evidence-informed policy making in health care. Previous research has reported essential partnership contextual factors and mechanisms that promote evidence-based policy making and practice in health care. However, the studies have not yet informed whether public care agency leaders’ and academic researchers’ perceptions of partnership purpose formulation and coalition building evolve through the PAP life cycle and whether public care agency leaders’ use of research evidence differs through life cycle stages.

**Objective:**

This exploratory study aims to focus on PAPs designed to improve youth mental health and well-being outcomes. This study also aims to identify public care agency leaders’ and academic researchers’ perceptions of PAP purpose formulation (structure, goals, primary function, and agenda-setting process) and coalition building (mutual benefits, trust, convener’s role, member role clarity, and conflict management) by PAP life cycle stage and examine whether public care agency leaders’ use of research evidence differs according to the perception of PAP purpose formulation and coalition building through the PAP life cycle.

**Methods:**

A web-based survey of PAP experience was conducted by recruiting academic researchers (n=40) and public care agency leaders (n=26) who were engaged in PAPs for the past 10 years. Public care agency leaders additionally participated in the survey of the Structured Interview for Evidence Use scale (n=48).

**Results:**

Most public care agency leaders and academic researchers in PAPs formed, matured, and sustained perceived their PAP as having purpose formulation context well aligned with their organizational purpose formulation context, pursuing mutual benefits, having leadership representation and role clarity, having a higher level of trust, and knowing how to handle conflicts. Most PAPs across all life cycle stages crystallized another issue to focus, but not all PAPs with issue crystallization had purpose reformulation. Public care agency leaders who trusted academic researchers in their PAP had greater use of research evidence. Public care agency leaders in PAPs that had gone through new issue crystallization also showed greater use of research evidence compared with those that had not.

**Conclusions:**

To promote public care agency leaders’ use of research evidence, focusing on developing trusting partnerships and continuously crystallizing PAP issues are important.

**International Registered Report Identifier (IRRID):**

RR2-10.2196/14382

## Introduction

### Background

Partnerships between academic institutions and public care agencies (public–academic partnerships [PAPs]) can promote effective public policy making and care delivery. For example, local US public health departments that formally partner with academic institutions are more likely than those not engaged in partnership with academic institutes to make evidence-based policy making and implement evidence-based interventions in health care delivery [1]. Previous studies have demonstrated the important role of PAPs in training service providers [2-5], supporting the implementation of promising evidence-based practices [3-7], and conducting systems evaluation that inform policy development and program planning [2,4]. Such partnerships have effectively responded to the need for additional, more diverse, and more inclusive mental health and child welfare services [2,3,8-10].

Although previous studies have demonstrated the positive impact of PAPs on youth mental health and well-being outcomes, few empirical studies have examined whether and how PAP contexts and mechanisms evolve through the PAP life cycle and which PAP contexts and mechanisms foster public agency leaders’ use of research evidence to improve youth mental health and well-being [11]. Public mental health and child welfare agencies are expected to increase the use of evidence-based care to improve mental health and well-being of vulnerable youth [12-16]. Many public care agencies partner with academic researchers to meet these expectations. Considering the multifaceted nature of public mental health and child welfare systems in the United States [17-20], it is important to develop a better understanding of the context and mechanisms that promote successful PAPs and evidence use by policy makers to improve youth outcomes.

This study has 3 aims. First, we describe a new integrated framework to understand PAP development through the PAP life cycle and potential PAP contexts and mechanisms that foster public care agency leaders’ use of research evidence. Second, we summarize the literature to provide empirical support for the integrated framework, focusing on the contexts and mechanisms of PAP purpose formulation and coalition building. Third, we report our study that comprehensively explored the relationship between PAP purpose formulation and coalition building and public care agency leaders’ use of research evidence by PAP life cycle stages of formed, matured, sustained, declining, and terminated.

### Key PAP Process

Although research on individual components of the partnership process has revealed important information about factors that support successful partnerships, the literature has yet to bring these components together into an integrated framework [11]. Such a framework would offer a way to examine the totality of PAPs, including the contexts in which they initiate and mature, the mechanisms that propel them forward, and the outcomes that they define and achieve at various stages in relation to public care agency leaders’ use of research evidence. The integrated framework by Kang-Yi [11] introduces concrete components of partnership purpose formulation and coalition building as the key contexts and mechanisms of PAPs that lead to policy makers’ use of research evidence. The framework consists of three theoretical perspectives: the social partnerships perspective [21,22], the organizational life cycle perspective [23-26], and the realist evaluation perspective [27,28].

On the basis of the social partnerships and organizational life cycle perspectives [21,25], the integrated framework posits that PAPs that continuously reformulate partnership purposes and build coalitions are likely to successfully evolve through life cycle stages of being formed, matured, and sustained. Public care agency leaders in successful PAPs (being matured or sustained compared with being just formed, declining, or terminated) are more likely to use research evidence. According to the social partnerships perspective by Waddock [21], purpose formulation processes include identifying clear goals and the primary function of partnership, creating a partnership structure, and setting partnership agenda. Coalition building processes include pursuing mutual benefits for each partner, building trust among partners, solidifying the convener’s role, clarifying the roles of all parties, and managing conflict [21]. The realist evaluation perspective provides a methodology for configuring contexts, mechanisms, and outcomes to examine the interplay of partnership purpose formulation, coalition building, and public care agency leaders’ use of research evidence in each PAP life cycle stage and overall evolvement of PAP [27-29]. The integrated framework emphasizes continuous purpose formulation and coalition building to adjust to changing partnership environment, sustain PAP, and promote public care agency leaders’ use of research evidence.

### Purpose Formulation

#### Agenda-Setting

One key ingredient in successful PAPs is the development of a clear purpose formulation among partners. Focusing on the needs of policy makers [2,6,30-32] and having public care agency representatives who are also skilled researchers driving the agenda-setting process are important [2].

#### Goals

Setting clear goals for a PAP is an important aspect of achieving and measuring success. Clear PAP goals have the power to keep partners focused on working toward positive outcomes [10]. PAPs in which goals are aligned with the goals of each partnering entity can contribute to the success and longevity of those PAPs [4-6]. PAPs with clear goals can promote the use of evidence by policy makers [30].

#### Primary Function

PAPs can play diverse primary functions, including generating knowledge related to the development and implementation of evidence-based policy making and practices, generalizing practices to a larger population, disseminating knowledge related to the implementation of evidence-based practices, and offering technical assistance, such as professional training and program evaluation in improving service quality and outcomes [11]. Given that public care agencies and academic institutes pursue diverse missions and primary functions, alignment in primary functions between a public care agency and an academic research institute can influence PAP sustainability and use of evidence by policy makers.

#### Structure

A partnership structure involves shaping governance processes, agreements around dissemination of findings, data sharing, business arrangements, ethics approvals, determining partnership mission, and general coordination among the partners [5,7,31-34]. The degree and quality of formalized structure shapes the extent of PAP success [5,32,35].

### Partnership Coalition Building

Previous studies have shown that coalition building, including mutual benefits and trust, plays a critical role in successful partnerships and in promoting the use of evidence by policy makers. The key dimensions of partnership coalition building include mutual benefits, trust, convener’s role, member role clarity, and conflict management.

#### Mutual Benefits

Successful PAPs are found to pursue mutual benefits, such as having specific agreement that ensures strategic advantages for both parties, smoother facilitation of contracts, financial incentives for the university, conducting actionable research, offering innovative ideas, improving the quality of services, offering researchers the benefit of evaluating a new theoretical model, and facilitating knowledge translation to direct practice [10,30,34-36]. Although PAPs offer a range of mutual benefits, they are not without risk. For example, time, effort, and cost of work are costs for all parties involved [36]. Risks specific to researchers include opportunity costs of spending time on projects that may not lead to publications and the potential negative impact of a changing political environment [34,36]. Risks for policy makers include spending social capital to justify engagement in the PAP, working with researchers who might not appreciate the complexity involved in the PAP work, the potential that research outcomes might not be practical, and the unknown impact the partnership may have on the organization [34,36].

#### Trust

Trust is another vital component of partnerships’ success. Trust plays a key role in the sustainability of partnerships, leading to continued work, additional projects, and system-level changes. Trusting relationships among partners also support PAPs in weathering leadership changes, particularly when that work has become integral to the functioning of an agency, promoting more efficient and purposeful engagement of policy makers in the research process [31,36]. Trust among partners may also facilitate the use of evidence by policy makers. For example, some PAPs appoint personnel specifically to serve as relationship cultivators and to seek input into research questions to be explored by PAPs [30].

#### Convener’s Role

PAPs need conveners to bring partners together into a partnership formation. Previous studies have documented the importance of such a role in bringing partners together in long-standing relationships within both organizations, identifying problem areas and developing initiatives in response, maintaining the necessary structure of PAP to disseminate the information generated by the partnership, and promoting the use of research evidence by policy makers [33,35,37]. Individuals who possess knowledge spanning both research and policy realms can support translating knowledge into the policy process [35].

#### Role Clarity

Clear delineation of roles among partners related to developing research questions and methodology as well as the eventual dissemination of the findings is important for successful PAPs [30,33]. In addition, clear communication between partners about how decisions are to be made and whether researchers can provide policy recommendations is critical [10], as these decisions can make a difference in informing policy makers and promoting the use of research evidence among policy makers. Partnerships that are slow in building comprehensive leadership teams and having members who are unsure of their roles can delay the generation of useful evidence for policy.

#### Conflict Management

Conflict is not unusual in the life of a partnership. Disagreement over project aims and funding [30] and other partnership processes, such as agenda-setting and contracting, can increase. Effective conflict management skills are important in building successful PAPs that lead to the use of research evidence in policy making.

### This Study: Web-Based Survey of PAPs

Our study aims to focus on PAPs designed to improve youth mental health and well-being. This study also aims to identify whether contexts and mechanisms of PAP partnership purpose formulation (structure, goals, and primary function as contexts and agenda-setting process as mechanism) and coalition building (convener’s role, leadership representation, role clarity, and conflict management as contexts and mutual benefits and trust as mechanisms) evolve through PAP life cycle stages (formed, matured, sustained, declining, and terminated). We also examined whether public care agency leaders’ use of research evidence differs according to their perception of the PAP life cycle stage, purpose formulation, and coalition building. Research evidence was defined as relevant conceptual frameworks or reviews and empirical findings from systematic qualitative, quantitative, or mixed research methods projects [38]. The study was approved by the institutional review board of the University of Pennsylvania (see Kang-Yi [11] for the published study protocol).

## Methods

### Sampling and Participant Recruitment

A web-based survey of PAP partnership experience and use of research evidence was conducted by recruiting academic researchers and public care agency leaders who were engaged in PAPs. See Page et al [39] for a detailed discussion of the approach used to identify PAP researchers and public care agency leaders. To recruit public care agency leaders and academic researchers who were engaged in PAPs, we identified PAPs through two primary methods: a web-based search of peer-reviewed journals and Google for key terms related to youth-focused PAPs and national and local meetings of professionals and researchers in the fields of mental health and child welfare. A total of 87 PAPs were identified, which met the following criteria: formed on a project, program, or intervention basis or as a consortium; aimed to improve mental health and well-being outcomes for youth aged 12-25 years; and comprised at least one or more state or local county mental health and child welfare agencies and one or more academic researchers. PAPs focused on youth outside the United States or established outside the United States and PAPs terminated within 10 years before the study initiation in 2017 were excluded. Of the 87 PAPs identified, we reached out to at least one public care agency leader in 67 PAPs and at least one academic researcher in 83 PAPs.

Once we identified researchers and public care agency leaders, we emailed them a link to the web-based survey along with introductory information about the nature of the study. A US $35 gift card was offered for full completion of a survey. Respondents were informed that the link was unique to them and asked not to share it with others. Data were collected from March 2019 to February 2020. The survey was tested for usability and accuracy by the research team and a small number of colleagues before being shared with potential respondents. In addition, the Checklist for Reporting Results of Internet E-Surveys [40] was used to report the survey as needed.

### Survey Measures

To respond to the survey questionnaire, the participants were asked to focus on the latest PAP or one of the PAPs for the past decade if they were not engaged in PAP at the time of the survey. The Structured Interview for Evidence Use (SIEU) [41] was used to identify public care agency leaders’ engagement level of research evidence, which refers to the frequency of using various types of sources for research evidence; public care agency leaders’ ratings of the importance of evaluating the validity, reliability, and relevance of research evidence; and various factors leading public care agency leaders to use or ignore research evidence in deciding to adopt a new program or intervention. The SIEU was developed based on the posit that research use is driven by context and social relationships [41]. Thus, SIEU as a tool reflects the integrated conceptual framework being tested in this study. SIEU includes input, process, and output scales. The input scale (20 items) assesses the source of research evidence that public care agency leaders obtain. The process scale assesses how public care agency leaders evaluate the research evidence obtained and includes 3 subscales of self-assessment for validity and reliability of research evidence (10 items), reliance on others (5 items), and self-assessment for relevance (5 items). The output scale (20 items) assesses whether public care agency leaders eventually use the research evidence or ignore the evidence. The measurement responses use a 5-point Likert-type scale ranging from 1 (not at all) to 5 (all the time) for the items contained in the input scale and a 5-point Likert-type scale ranging from 1 (not important) to 5 (very important) for the items contained in the process and output scales. Each subscale measure and the total SIEU score are represented as average scores. Higher scores indicate higher agreement with the sources of evidence obtained for the input scale, more frequent evaluation of research evidence for the process scale, and greater use of research evidence for the output scale. SIEU has shown high internal consistency reliability (Cronbach α=.88) [41].

The PAP experience survey was developed for this study [11]. The questionnaire included 41 questions that were based on the potential PAP context, mechanism, and outcome configuration developed for the study [11,39]. These questions included both a Likert-type scale and open-ended questions. The survey items focused on the following four areas: (1) partnership purpose formulation (structure, goals, primary function, and agenda-setting process), (2) perceptions of partnership coalition building (mutual benefit, trust, convener’s role, leadership representation, role clarity, and conflict management), (3) perception of the PAP life cycle stage, and (4) public care agency leaders’ use of research evidence. We built and administered the web-based survey in the Research Electronic Data Capture [42], a secure web-based data collection tool that includes data entry forms and web surveying features.

A total of 48 public care agency leaders participated in the web-based SIEU survey scale [41], and 40 academic researchers and 26 public care agency leaders participated in the PAP experience survey. The survey response rates were 72% (48/67) for the SIEU survey, 48% (40/83) for academic researchers’ PAP experience survey, and 39% (26/67) for public care agency leaders’ PAP experience survey.

### Analysis

The reliability of the SIEU was calculated using Cronbach α internal consistency for each of the subscales and the overall scale. Frequencies, percentages, and mean scores were calculated to identify (1) public care agency leaders’ and academic researchers’ ratings of alignment between PAP structure, goals, primary function, and agenda-setting process and their organizational structure, goals, primary function, and agenda-setting process by PAP life cycle stage (formed, matured, sustained, declining, and terminated); (2) public care agency leaders’ and academic researchers’ ratings of PAP coalition building (mutual benefits, trust, convener’s role, leadership representation, role clarity, and conflict management) by PAP life cycle stage; (3) public care agency leaders’ and academic researchers’ ratings of their partnership outcomes (identifying another issue to focus on and reformulate PAP purpose); and (4) public care agency leaders’ use of research evidence by the ratings of PAP life cycle stage, purpose formulation, and coalition building.

The original study design [11] was to recruit academic researchers and public care agency leaders in pairs. However, because of the low response rate for the PAP experience survey, we conducted group-level analysis for public care agency leaders and academic researchers, respectively, instead of conducting the analysis in pairs.

## Results

### Demographic Characteristics and Work Experience of Study Participants

As shown in Multimedia Appendix 1, public care agency leaders’ age and years of experience in the fields were distributed evenly. Of the public care agency leaders who answered the demographics and work experience questions, most (20/31, 65%) held a master’s degree. More than two-thirds of the public care agency leaders were women (21/31, 68%) and White (22/31, 71%). More than two-thirds of public care agency leaders (21/31, 68%) reported being at their current organizations for more than 10 years. More than three-fourths of public care agency leaders (24/31, 77%) had been involved in their current PAP for fewer than 10 years. The PAP roles they played were diverse and distributed evenly, and most (23/31, 74%) of public care agency leaders reported having been engaged in 5 or fewer PAPs.

As shown in Multimedia Appendix 2, academic researchers’ age and years of experience were also evenly distributed as were years at the current organization. Of the academic researchers who answered the demographic and work experience questions, most were women (30/40, 75%) and White (33/40, 83%) and held a doctoral degree (26/40, 65%). More than one-fourth of the academic researchers (11/40, 28%) had been involved with their current PAP for more than 10 years. The PAP roles they played were diverse, and only under one-third (11/40, 28%) identified their role as principal investigator, lead evaluator, and university lead.

### PAP Life Cycle Stages and SIEU Scale Scores

The average total SIEU score was 3.1 (SD 0.81; range 0.9-4.1). The internal consistency reliability of the SIEU based on the study sample was high (Cronbach α=.89). The mean score for the SIEU Input scale, the assessment of the source of research evidence that public care agency leaders obtain, was 2.9 (SD 0.46; range 1.8-3.9). The internal consistency reliability of the input scale was a Cronbach α value of .80. The mean SIEU process scale, the assessment of how public care agency leaders evaluate research evidence obtained, was 3.8 (SD 0.68; range 0.4-4.8). The internal consistency reliability of the process scale was a Cronbach α value of .85. The mean SIEU output scale, the assessment of public care agency leaders’ use of research evidence, was 3.1 (SD 0.74; range 0.2-3.9). The internal consistency reliability of the output scale was a Cronbach α value of .74.

**Figure 1:**
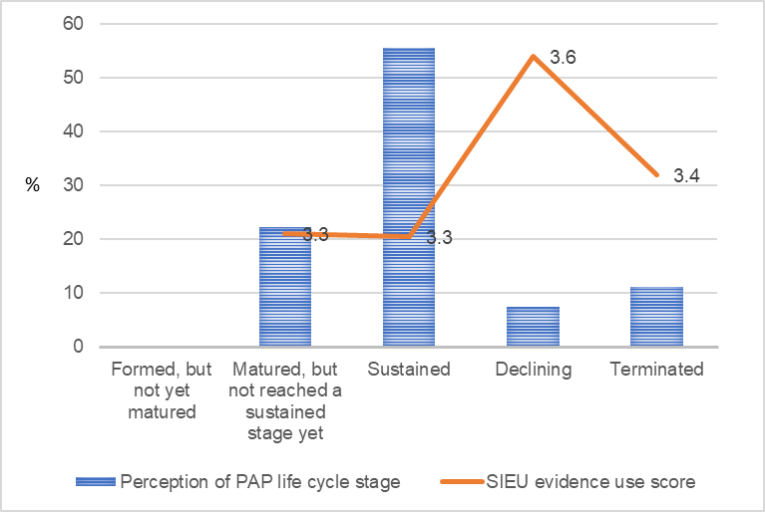
Public care agency leaders’ perception of public–academic partnership life cycle stage and the Structured Interview for Evidence Use score. PAP: public–academic partnership; SIEU: Structured Interview for Evidence Use.

As shown in Figure 1, 56% (15/26) of the public care agency leaders answered that their PAP was in a sustained stage, 22% (6/26) answered that their PAP was matured but did not reach a sustained stage yet, 11% (3/26) answered that their PAP was terminated at the time of the survey, 7% (2/26) answered that their PAP was declining, and 4% (1/26) answered that they were unsure about the stage of their PAP life cycle. None of the public care agency leaders answered that their PAP was formed but not matured yet.

For academic researchers, 45% (18/40) of the academic researchers answered that their PAP was in a sustained stage, 18% (7/40) answered that their PAP was matured but did not reach a sustained stage yet, 18% (7/40) answered that their PAP was formed but not reached a matured stage yet, 10% (4/40) answered that their PAP was declining, and another 10% (4/40) answered that their PAP was terminated.

For public care agency leaders’ use of research evidence (Figure 1), the public care agency leaders who answered that their PAP was declining had the highest SIEU output score (mean score 3.6, SD 0.42), followed by those who answered that their PAP was terminated (mean score 3.4, SD 0.13), those who answered that their PAP was mature but did not reach a sustained stage yet (mean score 3.3, SD 0.24), and those who answered that their PAP was sustained (mean score 3.3, SD 0.32).

### Perceptions of Purpose Formulation Context and Mechanism (Primary Function, Goals, Structure, and Agenda-Setting Process) and PAP Life Cycle Stage

As shown in Table 1, for PAPs in matured and sustained stages, only one public care agency leader in each group (1/6, 17% and 1/7, 7% of the PAPs, respectively) perceived the primary function of their PAP as perfectly aligned with the primary function of their organization. None of the public care agency leaders in PAPs declining and PAPs terminated perceived perfect alignment. A total of 4 academic researchers in formed, matured, and sustained PAPs (1/7, 14%; 1/7, 14%; and 2/17, 12% of the PAPs, respectively) perceived the primary function of partnership as perfectly aligned with the primary function of their organization.

Regarding the alignment of structures between PAP and partnering organizations, more than 86% (57/66) of both public care agency leaders and academic researchers answered that the structures were fairly well to perfectly aligned across all PAP life cycle stages. Three of the academic researchers in declining and terminated PAPs (2/4, 50% and 1/4, 25% of the PAPs, respectively) perceived very little alignment in the structures, whereas all public care agency leaders in PAPs declining and terminated perceived very well or perfectly well-aligned structures. All public care agency leaders perceived their PAP goals as fairly well to perfectly aligned with their organizational goals across all PAP life cycle stages. On the other hand, 2 of the academic researchers in PAPs declining and terminated (2/8, 25% of the PAPs) perceived their PAP goals as very little aligned with their organizational goals.

As shown in Table 2, 3 of the 5 public care agency leaders in the PAPs declining and terminated, perceived their PAP agenda-setting process as not at all or very little driven by the public care agency leaders. Academic researchers’ perception was similar; 3 of the 15 academic researchers in the PAPs formed, declining, and terminated perceived their PAP agenda-setting process as not at all or very little driven by public care agency leaders. More than 97% (30/31) of academic researchers in formed, matured, and sustained PAPs perceived their PAP agenda-setting process as driven by public care agency leaders. Almost half of the public care agency leaders (n=12) perceived very little of their PAP agenda process as driven by academic researchers, and this was consistent regardless of their perception of the PAP life cycle stage. The academic researchers’ perceptions were similar. Regardless of the PAP life cycle stage, almost half of academic researchers (n=18) perceived their PAP agenda-setting as not at all or very little driven by the researcher.

**Table 1:** Public–academic partnership purpose formulation context: perception of alignment in primary function, structure, and organizational goals (public care agency leaders [N=26] and academic researchers [N=40])^a^.

Parameters			Formed, n (%)					Matured, n (%)				Sustained, n (%)			Declining, n (%)					Terminated, n (%)		
			Public care agency leaders (n=0)	Academic researchers (n=7)			Public care agency leaders (n=6)		Academic researchers (n=7)		Public care agency leaders (n=15)		Academic researchers (n=17)	Public care agency leaders (n=2)		Academic researchers (n=4)			Public care agency leaders (n=3)		Academic researchers (n=4)	
**Primary function^b^**																						
	Not at all	N/A^c^			0 (0)	0 (0)				0 (0)	0 (0)		0 (0)	0 (0)			0 (0)	0 (0)				1 (25)
	Very little	N/A			0 (0)	0 (0)				0 (0)	0 (0)		0(0)	0 (0)			1 (25)	0 (0)				0 (0)
	Fairly well	N/A			2 (29)	2 (33)				0 (0)	3 (20)		2 (12)	0 (0)			2 (50)	1 (33)				0 (0)
	Quite well	N/A			3 (43)	0 (0)				3 (42)	2 (13)		4 (24)	1 (50)			0 (0)	2 (67)				1 (25)
	Very well	N/A			1 (14)	3 (50)				3 (42)	7 (47)		9 (53)	1 (50)			1 (25)	0 (0)				2 (50)
	Perfectly	N/A			1 (14)	1 (17)				1 (14)	1 (67)		2 (12)	0 (0)			0 (0)	0 (0)				0 (0)
	Do not know or unsure	N/A			0 (0)	0 (0)				0 (0)	1 (67)		0 (0)	0 (0)			0 (0)	0 (0)				0 (0)
**Structure alignment^d^**																						
	Not at all	N/A			1 (14)	0 (0)				0 (0)	0 (0)		0 (0)	0 (0)			0 (0)	0 (0)				0 (0)
	Very little	N/A			1 (14)	1 (17)				0 (0)	1 (67)		0 (0)	0 (0)			2 (50)	0 (0)				1 (25)
	Fairly well	N/A			1 (14)	2 (33)				1 (14)	5 (33)		2 (12)	0 (0)			1 (25)	0 (0)				1 (25)
	Quite well	N/A			2 (29)	0 (0)				4 (57)	3 (20)		5 (29)	1 (50)			1 (25)	1 (33)				1 (25)
	Very well	N/A			2 (29)	3 (50)				1 (14)	5 (33)		8 (47)	1 (50)			0 (0)	2 (67)				1 (25)
	Perfectly	N/A			0 (0)	0 (0)				0 (0)	1 (67)		2 (12)	0 (0)			0 (0)	0 (0)				0 (0)
	Do not know or unsure	N/A			0 (0)	0 (0)				1 (14)	0 (0)		0 (0)	0 (0)			0 (0)	0 (0)				0 (0)
**Organizational goals^e^**																						
	Not at all	N/A			0 (0)	0 (0)				0 (0)	0 (0)		0 (0)	0 (0)			0 (0)	0 (0)				0 (0)
	Very little	N/A			0 (0)	0 (0)				0 (0)	0 (0)		0 (0)	0 (0)			1 (25)	0 (0)				1 (25)
	Fairly well	N/A			2 (29)	1 (17)				0 (0)	4 (27)		2 (12)	0 (0)			2 (50)	1 (33)				0 (0)
	Quite well	N/A			1 (14)	1 (17)				3 (43)	2 (13)		3 (18)	1 (50)			0 (0)	1 (33)				1 (25)
	Very well	N/A			3 (43)	4 (67)				3 (43)	8 (53)		10 (59)	1 (50)			1 (25)	1 (33)				2 (50)
	Perfectly	N/A			1 (14)	0 (0)				1 (14)	1 (67)		2 (12)	0 (0)			0 (0)	0 (0)				0 (0)
	Do not know or unsure	N/A			0 (0)	0 (0)				0 (0)	0 (0)		0 (0)	0 (0)			0 (0)	0 (0)				0 (0)

^a^For each cell, the within-column percentages of public care agency leaders’ and academic researchers’ perceptions are presented.

^b^Response missing for public care agency leaders (n=1); response missing for academic researchers (n=1).

^c^N/A: not applicable.

^d^Response missing for public care agency leaders (n=0); response missing for academic researchers (n=1).

^e^Response missing for public care agency leaders (n=0); response missing for academic researchers (n=1).

**Table 2:** Public–academic partnership (PAP) purpose formulation mechanism (agenda-setting process; public care agency leaders [N=26] and academic researchers [N=40])^a^.

Parameters			Formed, n (%)				Matured, n (%)				Sustained, n (%)			Declining, n (%)				Terminated, n (%)	
			Public care agency leaders (n=0)		Academic researchers (n=7)		Public care agency leaders (n=6)		Academic researchers (n=7)		Public care agency leaders (n=15)		Academic researchers (n=17)	Public care agency leaders (n=2)		Academic researchers (n=4)	Public care agency leaders (n=3)		Academic researchers (n=4)
**Perception of PAP agenda driven by researchers^b^**																			
	Not at all	N/A^c^		0 (0)		1 (17)		1 (14)		3 (20)		2 (12)			0 (0)	0 (0)	1 (33)		1 (25)
	Very little	N/A		3 (43)		1 (17)		1 (14)		4 (27)		6 (35)			1 (50)	3 (75)	1 (33)		1 (25)
	Fairly well	N/A		2 (29)		2 (33)		0 (0)		2 (13)		2 (12)			0 (0)	0 (0)	0 (0)		1 (25)
	Quite well	N/A		2 (29)		1 (17)		2 (29)		1 (7)		3 (24)			1 (50)	1 (25)	0 (0)		0 (0)
	Very well	N/A		0 (0)		1 (17)		3 (43)		3 (20)		1 (6)			0 (0)	0 (0)	1 (33)		1 (25)
	Perfectly	N/A		0 (0)		0 (0)		0 (0)		0 (0)		1 (6)			0 (0)	0 (0)	0 (0)		0 (0)
	Do not know or unsure	N/A		0 (0)		0 (0)		0 (0)		0 (0)		1 (6)			0 (0)	0 (0)	0 (0)		0 (0)
**Perception of PAP agenda driven by public care agency leaders^d^**																			
	Not at all	N/A		0 (0)		0 (0)		0 (0)		0 (0)		0 (0)			0 (0)	0 (0)	0 (0)		1 (25)
	Very little	N/A		1 (14)		0 (0)		0 (0)		0 (0)		0 (0)			1 (50)	2 (50)	1 (33)		0 (0)
	Fairly well	N/A		0 (0)		1 (17)		1 (14)		6 (40)		3 (18)			0 (0)	2 (50)	1 (33)		1 (25)
	Quite well	N/A		4 (57)		3 (50)		5 (71)		3 (20)		5 (29)			1 (50)	0 (0)	1 (33)		1 (25)
	Very well	N/A		2 (29)		2 (33)		1 (14)		5 (33)		7 (41)			0 (0)	0 (0)	0 (0)		1 (25)
	Perfectly	N/A		0 (0)		0 (0)		0 (0)		1 (7)		2 (12)			0 (0)	0 (0)	0 (0)		0 (0)
	Do not known unsure	N/A		0 (0)		0 (0)		0 (0)		0 (0)		0 (0)			0 (0)	0 (0)	0 (0)		0 (0)

^a^For each cell, within-column percentages of public care agency leaders’ and academic researchers’ perception are presented, respectively.

^b^Response missing for public care agency leaders, (n=2); response missing for academic researchers (n=1).

^c^N/A: not applicable.

^d^Response missing for public care agency leaders, (n=0); response missing for academic researchers (n=1).

### PAP Coalition Building Context (Convener’s Role, Leadership Representation, Role Clarity, and Conflict Management) and PAP Life Cycle Stage

As shown in Table 3, most public care agency leaders and academic researchers in PAPs formed, matured, and sustained had a convener who gathered people together to carry out partnership processes, such as issue crystallization, partnership coalition building, and agenda-setting. In total, 3 of the 8 academic researchers in PAPs declining and terminated (2/4, 50% and 1/4, 25% of the PAPs, respectively) and 1 public care agency leader (1/3, 33%) in PAPs terminated answered that their PAPs were missing a convener.

Public care agency leaders’ perceptions of clear leadership representation and role clarity did not differ according to the PAP life cycle stage. Approximately 27% (4/15) of public care agency leaders in PAPs sustained answered that their PAP rarely or only occasionally had leadership representation and role clarity. Overall, 30 academic researchers (30/40, 75% of all academic researchers) answered that their PAP always had leadership representation and clear roles.

Most public care agency leaders and academic researchers answered that they experienced partnership conflict across all PAP life cycle stages, except for PAPs in a formed stage. Most public care agency leaders (up to 23/26, 88%; range 67%-100% across all PAP life cycle stages) answered that PAP members knew how to manage partnership conflicts. Most academic researchers in PAPs formed, matured, and sustained (up to 29/31, 94%; range 86%-100%) answered that their PAP members knew how to handle partnership conflicts. In total, 3 of the 8 academic researchers in PAPs declining and terminated answered that their PAP members knew how to handle partnership conflicts.

As shown in Table 4, public care agency leaders’ trust in researchers, academic researchers’ trust in public care agency leaders, and perception of pursuing mutual benefit in partnership agenda-setting did not show meaningful patterns by the PAP life cycle stage. Researchers’ perception of PAPs pursuing mutual benefit in partnership agenda-setting differed according to the PAP life cycle stage. Most researchers (30/31, 97%) in PAPs formed, matured, and sustained perceived their PAP very frequently or always pursuing mutual benefit and used to pursue mutual benefit in setting partnership agenda. PAPs sustained had the highest percentage of academic researchers (6/17, 35%) answering their PAP always pursued mutual benefits.

As shown in Table 5, across all PAP life cycle stages, most public care agency leaders and researchers answered that their PAP resulted in focusing on another issue. Academic researchers’ perception of their partnership leading to focus on another issue was the highest among PAPs matured (6/7, 86%), followed by PAPs sustained (14/17, 82%), PAPs declined (3/4, 75%), PAP terminated (2/4, 50%), and PAPs formed (3/7, 43%). More than two-thirds of the researchers (4/5, 67%) in PAPs matured answered that focusing on a new issue led to reformulating the PAP agenda-setting process. The majority of public care agency leaders (10/16, 63%) answered that the new issue did not result in reformulating the PAP agenda-setting process.

**Table 3:** Public–academic partnership (PAP) coalition building context (convener’s role, leadership representation, role clarity, and conflict management; public care agency leaders [N=26] and academic researchers [N=40])^a^.

Parameters			Formed, n (%)					Matured, n (%)					Sustained, n (%)					Declining, n (%)					Terminated, n (%)		
			Public care agency leaders (n=0)		Academic researchers (n=7)		Public care agency leaders (n=6)			Academic researchers (n=7)		Public care agency leaders (n=15)			Academic researchers (n=17)		Public care agency leaders (n=2)			Academic researchers (n=4)		Public care agency leaders (n=3)			Academic researchers (n=4)
**Perception of PAP having a convener who plays the role of gathering people together^b^**																									
	Yes	N/A^c^		6 (86)		5 (83)			5 (83)		9 (60)			14 (82)		2 (100)			2 (50)		2 (67)			1 (25)	
	No	N/A		1 (14)		0(0)			1 (17)		3 (20)			3 (18)		0 (0)			2 (50)		1 (33)			1 (25)	
	Used to have	N/A		0 (0)		1 (17)			0 (0)		2 (13)			0 (0)		0 (0)			0 (0)		0 (0)			2 (50)	
	Do not know or unsure	N/A		0 (0)		0 (0)			0 (0)		1 (7)			0 (0)		0 (0)			0 (0)		0 (0)			0 (0)	
**Perception of having clear leadership representation and roles^d^**																									
	Rarely	N/A		0 (0)		0 (0)			0 (0)		2 (13)			1 (6)		0 (0)			0 (0)		0 (0)			1 (25)	
	Occasionally	N/A		0 (0)		0 (0)			0 (0)		2 (13)			2 (12)		0 (0)			2 (50)		0 (0)			2 (50)	
	Frequently	N/A		0 (0)		1 (17)			1 (14)		1 (7)			1 (6)		0 (0)			2 (50)		1 (33)			0 (0)	
	Very frequently	N/A		0 (0)		2 (33)			1 (14)		1 (7)			4 (26)		0 (0)			0 (0)		1 (33)			1 (25)	
	Always	N/A		7 (100)		3 (50)			5 (71)		8 (53)			9 (53)		2 (100)			0 (0)		1 (33)			0 (0)	
	Used to have	N/A		0 (0)		0 (0)			0 (0)		1 (7)			0 (0)		0 (0)			0 (0)		0 (0)			0 (0)	
	Do not know or unsure	N/A		0 (0)		0 (0)			0 (0)		0 (0)			0 (0)		0 (0)			0 (0)		0 (0)			0 (0)	
**Experience of PAP conflict^e^**																									
	Yes	N/A		1 (14)		3 (50)			4 (57)		10 (67)			8 (47)		1 (50)			4 (100)		2 (67)			4 (100)	
	No	N/A		6 (86)		2 (33)			3 (43)		4 (27)			9 (53)		1 (50)			0 (0)		1 (33)			0 (0)	
	Do not know or unsure	N/A		0 (0)		1 (17)			0 (0)		1 (7)			0 (0)		0 (0)			0 (0)		0 (0)			0 (0)	
**Perception of PAP members knowing how to handle PAP conflicts^f^**																									
	Yes	N/A		7 (100)		4 (67)			6 (86)		14 (93)			16 (94)		2 (100)			1 (25)		3 (100)			2 (50)	
	No	N/A		0 (0)		2 (33)			0 (0)		0 (0)			1 (6)		0 (0)			2 (50)		0 (0)			2 (50)	
	Do not know or unsure	N/A		0 (0)		0 (0)			1 (14)		1 (7)			0 (0)		0 (0)			1 (25)		0 (0)			0 (0)	

^a^For each cell, the within-column percentages of public care agency leaders’ and academic researchers’ perceptions are presented.

^b^Response missing for public care agency leaders (n=0); response missing for academic researchers (n=2).

^c^N/A: not applicable.

^d^Response missing for public care agency leaders (n=0); response missing for academic researchers (n=1).

^e^Response missing for public care agency leaders (n=0); response missing for academic researchers (n=1).

^f^Response missing for public care agency leaders (n=0); response missing for academic researchers (n=1).

**Table 4:** Public–academic partnership (PAP) coalition building mechanism (mutual benefit and trust in PAP agenda-setting; public care agency leaders [N=26] and academic researchers [N=40])^a^.

Parameters		Formed, n (%)		Matured, n (%)			Sustained, n (%)				Declining, n (%)				Terminated, n (%)		
		Public care agency leaders (n=0)	Academic researchers (n=7)	Public care agency leaders (n=6)	Academic researchers (n=7)		Public care agency leaders (n=15)		Academic researchers (n=17)		Public care agency leaders (n=2)		Academic researchers (n=4)	Public care agency leaders (n=3)			Academic researchers (n=4)
**Perception of mutual benefit in PAP agenda setting^b^**																	
	Rarely	N/A^c^	0 (0)	1 (17)	0 (0)	0 (0)		0 (0)		0 (0)		0 (0)		0 (0)		1 (25)	
	Occasionally	N/A	1 (14)	0 (0)	0 (0)	0 (0)		0 (0)		0 (0)		3 (75)		1 (33)		1 (25)	
	Frequently	N/A	1 (14)	0 (0)	3 (50)	5 (33)		3 (18)		0 (0)		0 (0)		0 (0)		2 (50)	
	Very frequently	N/A	2 (29)	0 (0)	2 (33)	2 (13)		8 (47)		0 (0)		1 (25)		1 (33)		0 (0)	
	Always	N/A	3 (43)	4 (67)	1 (17)	7 (47)		6 (35)		2 (100)		0 (0)		1 (33)		0 (0)	
	Used to pursue mutual benefit	N/A	0 (0)	1 (17)	0 (0)	1 (7)		0 (0)		0 (0)		0 (0)		0 (0)		0 (0)	
	Do not know or unsure	N/A	0 (0)	0 (0)	0 (0)	0 (0)		0 (0)		0 (0)		0 (0)		0 (0)		0 (0)	
**Perception of the level of trust academic researchers have for public care agency leaders^d^**																	
	High	N/A	5 (86)	4 (67)	5 (71)	7 (47)		14 (83)		1 (50)		1 (25)		2 (67)		1 (25)	
	Moderate	N/A	1 (14)	1 (17)	1 (14)	5 (33)		2 (17)		1 (50)		1 (25)		1 (33)		1 (25)	
	Low	N/A	0 (0)	0 (0)	0 (0)	0 (0)		0 (0)		0 (0)		2 (50)		0 (0)		1 (25)	
	Used to have high level of trust	N/A	0 (0)	0 (0)	0 (0)	2 (13)		0 (0)		0 (0)		0 (0)		0 (0)		1 (25)	
	Do not know or unsure	N/A	0 (0)	1 (17)	1 (14)	1 (7)		0 (0)		0 (0)		0 (0)		0 (0)		0 (0)	
**Perception of the level of trust public care agency leaders have for academic researchers^e^**																	
	High	N/A	5 (71)	5 (83)	5 (63)	7 (47)		15 (83)		1 (50)		0 (0)		2 (67)		1 (25)	
	Moderate	N/A	2 (29)	0 (0)	1 (13)	5 (33)		2 (17)		1 (50)		3 (75)		1 (33)		2 (50)	
	Low	N/A	0 (0)	0 (0)	0 (0)	1 (7)		0 (0)		0 (0)		1 (25)		0 (0)		1 (25)	
	Used to have high level of trust	N/A	0 (0)	0 (0)	1 (13)	2 (13)		0 (0)		0 (0)		0 (0)		0 (0)		0 (0)	
	Do not know or unsure	N/A	0 (0)	1 (17)	0 (0)	0 (0)		0 (0)		0 (0)		0 (0)		0 (0)		0 (0)	

^a^For each cell, the within-column percentages of public care agency leaders’ and academic researchers’ perceptions are presented.

^b^Response missing for public care agency leaders (n=0); response missing for academic researchers (n=2).

^c^N/A: not applicable.

^d^Response missing for public care agency leaders (n=0); response missing for academic researchers (n=2).

^e^Response missing for public care agency leaders (n=0); response missing for academic researchers (n=0).

**Table 5:** Public–academic partnership (PAP) purpose formulation and coalition building outcome (new issue to focus and reformulation of PAP agenda-setting process; public care agency leaders [N=26] and academic researchers [N=40])^a^.

Parameters		Formed, n (%)			Matured, n (%)			Sustained, n (%)			Declining, n (%)			Terminated, n (%)	
		Public care agency leaders (n=0)	Academic researchers (n=7)	Public care agency leaders (n=6)		Academic researchers (n=7)	Public care agency leaders (n=15)		Academic researchers (n=17)	Public care agency leaders (n=2)		Academic researchers (n=4)	Public care agency leaders (n=3)		Academic researchers (n=4)
**Perception of PAP leading to focus on another issue^b^**															
	Yes	N/A^c^	3 (43)	3 (50)		6 (86)	10 (67)		14 (82)	1 (50)		3 (75)	2 (67)		2 (50)
	No	N/A	4 (57)	2 (33)		1 (14)	4 (27)		2 (12)	1 (50)		1 (25)	1 (33)		2 (50)
	Do not know or unsure	N/A	0 (0)	1 (17)		0 (0)	1 (7)		1 (6)	0 (0)		0 (0)	0 (0)		0 (0)
**Perception of PAP leading to reformulate PAP agenda-setting process^d^**															
	Yes	N/A	1 (33)	2 (67)		4 (67)	4 (40)		5 (36)	0 (0)		0 (0)	0 (0)		1 (50)
	No	N/A	2 (67)	1 (33)		2 (33)	6 (60)		9 (64)	1 (100)		2 (67)	2 (100)		1 (50)
	Do not know or unsure	N/A	0 (0)	0 (0)		0 (0)	0 (0)		0 (0)	0 (0)		1 (33)	0 (0)		0 (0)

^a^For each cell, the within-column percentages of public care agency leaders’ and academic researchers’ perceptions are presented.

^b^Response missing for public care agency leaders (n=0); response missing for academic researchers (n=1).

^c^N/A: not applicable.

^d^Response missing for public care agency leaders (n=10); response missing for academic researchers (n=12).

### PAP Purpose Formulation and Coalition Building and Public Care Agency Leaders’ Use of Research Evidence

**Figure 2:**
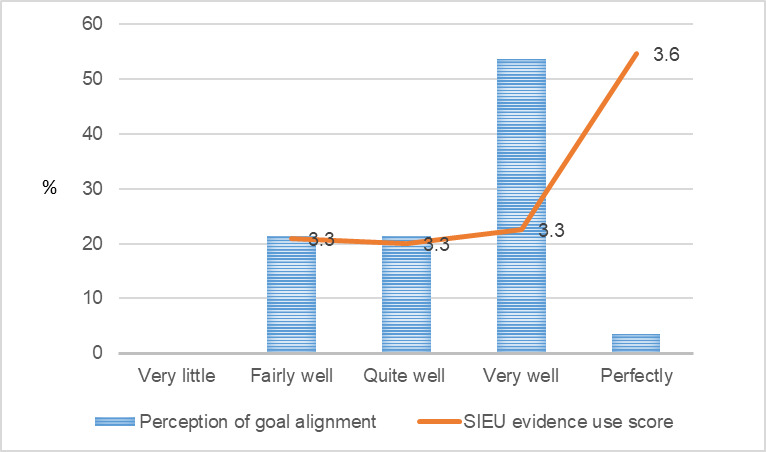
Public care agency leaders’ perception of goal alignment and the Structured Interview for Evidence Use score. SIEU: Structured Interview for Evidence Use.

**Figure 3:**
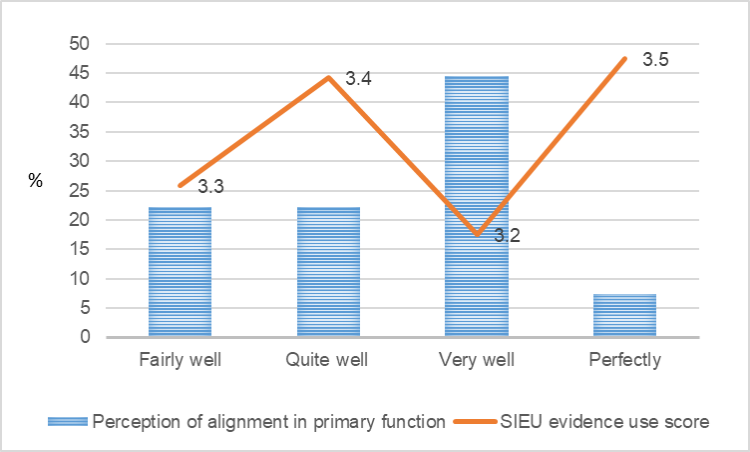
Public care agency leaders’ perception of primary function alignment and the Structured Interview for Evidence Use score. SIEU: Structured Interview for Evidence Use.

**Figure 4:**
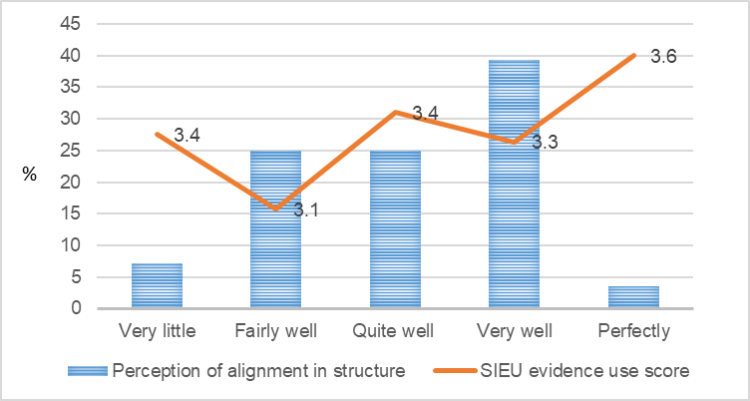
Public care agency leaders’ perception of structure alignment and the Structured Interview for Evidence Use score. SIEU: Structured Interview for Evidence Use.

**Figure 5:**
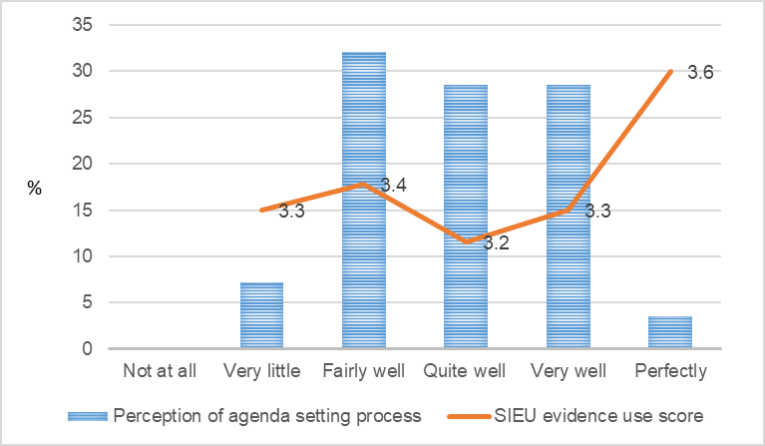
Public care agency leaders’ perception of agenda-setting driven by public care agency leaders and the Structured Interview for Evidence Use score. SIEU: Structured Interview for Evidence Use.

**Figure 6:**
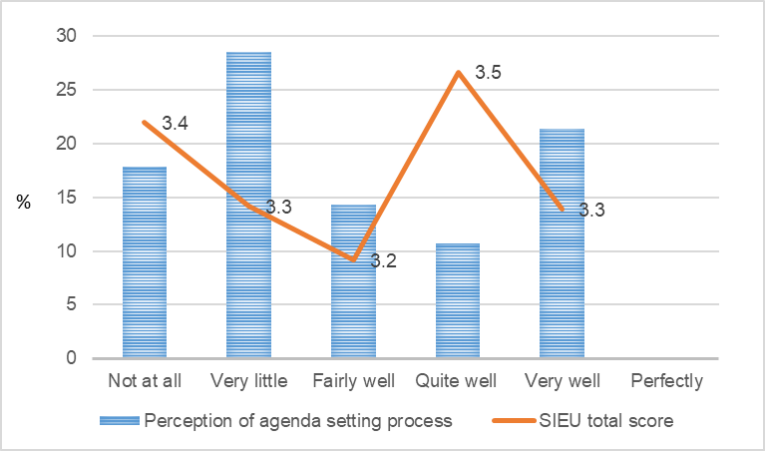
Public care agency leaders’ perception of agenda-setting driven by researchers and the Structured Interview for Evidence Use score. SIEU: Structured Interview for Evidence Use.

**Figure 7:**
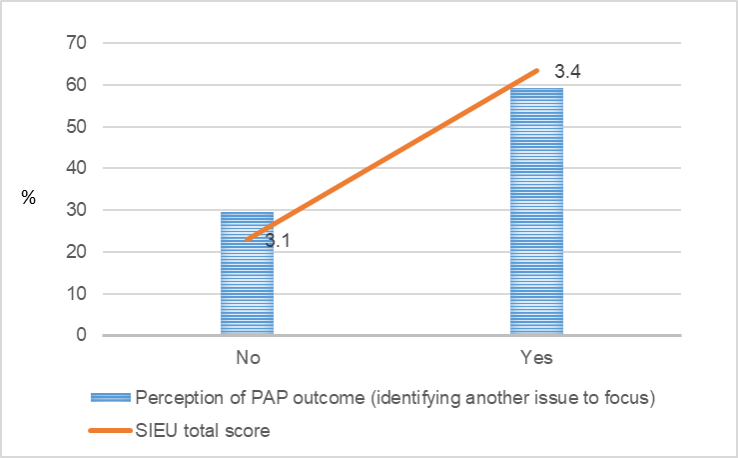
Public care agency leaders’ response for partnership issue crystallization and the Structured Interview for Evidence Use score. PAP: public–academic partnership; SIEU: Structured Interview for Evidence Use.

**Figure 8:**
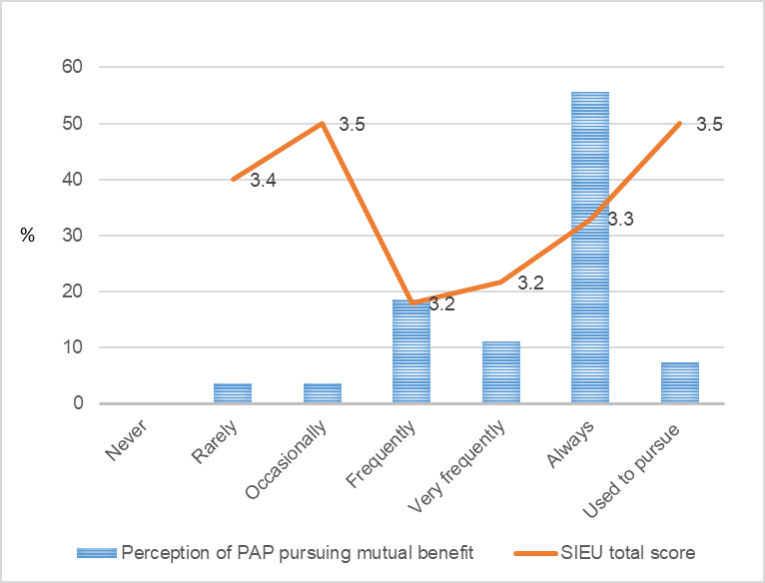
Public care agency leaders’ response for partnership pursuing mutual benefits and the Structured Interview for Evidence Use score. PAP: public–academic partnership; SIEU: Structured Interview for Evidence Use.

**Figure 9:**
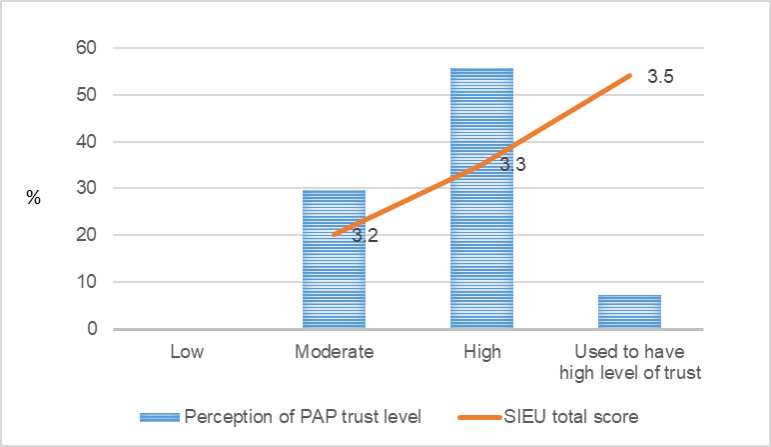
Public care agency leaders’ perception of level of trust researchers have for the public care agency leaders and the Structured Interview for Evidence Use score. PAP: public–academic partnership; SIEU: Structured Interview for Evidence Use.

Figures 1-9 present public care agency leaders’ perceptions of PAP purpose formulation and coalition building and their use of research evidence. The average SIEU output scale score that indicates public care agency leaders’ actual use of research evidence was the highest among the PAPs declining followed by PAPs terminated, PAPs formed, and PAPs matured (3.6, SD 0.42; 3.4, SD 0.13; 3.3, SD 0.32; and 3.3, SD 0.24, respectively). The average SIEU output scale score was higher in PAPs, which resulted in another issue to focus compared with the score of PAPs without issue recrystallization (SIEU scores 3.4, SD 0.28 vs 3.1, SD 0.25).

On the other hand, the SIEU output scale score did not show a correlated pattern with public care agency leaders’ perceptions of the agenda-setting process. Public care agency leaders who reported that their partnering researchers used to have trust in PAP leaders (public care agency leaders) showed the highest average SIEU output scale score (3.5, SD 0.21). The SIEU output scale scores did not show correlated pattern with the public care agency leaders’ perception of PAP seeking mutual benefit.

## Discussion

### Purpose Formulation, Coalition Building, and PAP Life Cycle Stages

The study findings show that overall, PAP purpose formulation including goals, primary function and structure, and partnership coalition building, including mutual benefits, trust, convener’s role, leadership representation, role clarity, and conflict management, are important contexts and mechanisms for PAPs to evolve through life cycle stages. For the partnership contexts and mechanisms, PAPs matured were perceived more positively than PAPs formed, and PAPs sustained were perceived more positively than PAPs matured by public care agency leaders and academic researchers. However, not all the contexts and mechanisms of purpose formulation and coalition building showed evolving through the PAP life cycle stages.

Most public care agency leaders and academic researchers in PAPs formed, matured, and sustained perceived the context of partnership purpose formulation as well aligned with those of their organization. Public care agency leaders and academic researchers in PAPs declining and terminated perceived a low level of alignment in the context. This echoes the findings from studies focused on PAPs in other fields, such as public health insurance [31], environmental health [32], health care delivery [30], child welfare and mental health services [10], and general and adult mental health care [2,4-6,37] in which successful PAPs were reported to have aligned structure, goals, and agenda-setting process.

More than 40% (30/66) of public care agency leaders and academic researchers in PAPs sustained perceived that PAP agenda-setting was not at all or little driven by them. Particularly, more than one-third of researchers perceived that their PAP agenda-setting was not at all driven by academic researchers. More than 50% (25/45) of public care agency leaders and academic researchers in PAP matured and sustained perceived their PAP as always having leadership presentation and role clarity. As demonstrated in previous studies [10,34], a continuous role clarity process that responds to changing environments and needs of the mental health, child welfare, and public health fields is important for PAPs to sustain. PAPs sustained are likely to have overcome periodic leadership shifts and changes in the political environment, successfully engaging new leaders in the partnership process and continuously clarifying the roles of the members of PAP [10].

Effective conflict management skills have been shown to be important in building successful PAPs in health care delivery [30]. Most public care agency leaders and researchers experienced partnership conflict regardless of the PAP life cycle stage, except for the researchers in PAPs formed. Most public care agency leaders and academic researchers in PAPs formed, matured, and sustained reported that their PAP members knew how to handle partnership conflicts.

We found that most PAPs across all life cycle stages crystallized another issue, but the issue of crystallization did not lead to purpose reformulation for most PAPs. Although partnerships are expected to constantly review and reformulate purpose and scan their environmental changes to increase their sustainability [21], it is possible that focusing on another issue does not require changes in the PAP agenda-setting process. We did not have information on whether the new issue crystallization required PAPs to reformulate partnership purpose. Further research on specific PAP context and mechanisms that result in PAP purpose reformulation will lead to gaining an in-depth understanding.

### Public Care Agency Leaders’ Use of Research Evidence by Perception of PAP Purpose Formulation, Coalition Building, and PAP Life Cycle Stage

Supporting the previous research [29] on context and mechanisms for successful PAPs, our study found that developing trusting relationships with public care agency leaders and continuously crystallizing PAP issues play an important role in not only increasing PAP sustainability but also fostering public care agency leaders’ use of research evidence. Public care agency leaders using research evidence may be more open to new ideas proposed by academic researchers and actively pursue issue crystallization. PAPs that continuously crystallize issues are also likely to lead public care agency leaders to be frequently exposed to research evidence. Public care agency leaders who reported their PAP as having a high level of trust in their partnering researchers also showed greater use of research evidence.

Unlike the previous research in health care delivery [30] that reports identifying clear and aligned goals as promoting partners’ prioritization of their work and eventual use of evidence, our study did not find greater use of research evidence among public care agency leaders who perceived their PAP goals, primary function, and structure well aligned with their organizational goals, primary function, and structure. Previous research on health care delivery [30] and public health [36] have reported a positive relationship between PAPs seeking mutual benefit and public care agency leaders’ use of research evidence. However, our study did not find the positive relationship. Public care agency leaders’ use of research evidence did not show a consistent pattern by the PAP life cycle stage. Public care agency leaders who perceived their PAP as declining showed the highest level of use of research evidence. This may be attributed to the small sample size, and further research is warranted. Future research with a larger study sample and mixed methods will provide further insights.

### Limitations

Our study has a few limitations. The number of public care agency leaders who participated in the PAP experience survey was limited to 26*.* We described in the informed consent that information provided by study participants would remain in a secure web-based database that only the key research staff could access, and that data would be analyzed at the aggregate level. Despite the statement of confidentiality and privacy protection written in the informed consent, the response rate from public care agency leaders was low. Some of the contexts and mechanisms of PAP purpose formulation and coalition building not varying by PAP life cycle stage may be attributed to the small sample size. Academic researchers’ and public care agency leaders’ PAP partnership experience were not analyzed in pairs because of the small sample size. Thus, our findings do not reflect the concordance level in the perception of academic researchers and public care agency leaders in pairs. The study findings may reflect social desirability bias from the respondents. For example, as noted by Ross et al [36], researchers may have reported on positive aspects of the relationships with public care agency leaders to avoid damaging connections, and policy makers might have reported stronger reliance on evidence use because of public emphasis on evidence use. Some of the PAP contexts, such as funding opportunities and mental health and child welfare policies at the federal and state levels, are expected to influence PAP sustainability and public care agency leaders’ use of research evidence. In this study, we focused on the contexts and mechanisms that can be applied to all PAPs in the fields instead of reviewing and interpreting PAP-specific contexts. A case analysis that incorporates PAP-specific contexts along with the purpose formulation and partnership coalition building can provide in-depth insights.

### Conclusions

Understanding factors that promote successful PAPs and evidence use by policy makers has the potential to improve outcomes for vulnerable youth populations served by public mental health and child welfare systems in the United States. PAPs declining can revive through making changes to adapt to continuously changing environment. Our study findings suggest that continuous trust cultivation through ongoing and clear communication and continuous issue crystallization may promote public care agency leaders’ use of research evidence. Academic researchers’ efforts to build trust with public care agency leaders and constantly formulate issues to meet the needs of public care agency leaders who constantly experience changes in the public care environment are essential. To promote mutual benefits that link to the use of research evidence, public care agencies should establish clear research and evaluation guidelines to inform researchers of expectations when initiating and forming PAPs.

Few studies have examined PAPs in the mental health and child welfare fields despite the frequent use of PAPs. Recently, there has been increased attention to PAPs in other related fields such as health care, with rapid advancement of science such as health information technology [43]. PAPs play an important role in translating research findings into innovative policies and practices. We urge academic researchers and public care agency leaders in the fields of mental health and child welfare to pay greater attention to further understanding the partnership context and mechanisms that promote innovative evidence-based policy and practice.

## Appendix

Multimedia Appendix 1 Public care agency leaders’ demographics and work experience.

Multimedia Appendix 2 Academic researchers’ demographics and work experience.

## Abbreviations

PAP: public–academic partnership
SIEU: Structured Interview for Evidence Use
