# Supplementary material for: Purpose Formulation, Coalition Building, and Evidence Use in Public–Academic Partnerships: Web-Based Survey Study
Source: JMIR Hum Factors. 2022 Jan 5;9(1):e29288. doi: 10.2196/29288 (PMC8771345; doi:10.2196/29288)
Supplement: Multimedia Appendix 1 [file humanfactors_v9i1e29288_app1.docx]

| **Public care agency leaders’ (N=31) age in years (%)** | |
| --- | --- |
| Less than 30 | 6.5 |
| 30-39 | 19.4 |
| 40-49 | 22.6 |
| 50-59 | 29.0 |
| 60 or older | 3.2 |
| “N/A” | 3.2 |
| Missing | 16.1 |
| **Public care agency leaders’ (N=31) years of experience in the field (%)** | |
| Fewer than 10 | 19.4 |
| 10-19 | 25.8 |
| 20-29 | 19.4 |
| 30-39 | 19.4 |
| 40 or more | 3.2 |
| Missing | 12.9 |
| **Public care agency leaders’ (N=31) years in the current organization (%)** | |
| Fewer than 10 | 45.2 |
| 10-19 | 19.4 |
| 20-29 | 9.7 |
| 30-39 | 12.9 |
| Missing | 12.9 |
| **Public care agency leaders’ (N=31) years involved with current PAP (%)** | |
| Fewer than 10 years | 77.4 |
| 10 or more years | 6.5 |
| Unspecified | 3.2 |
| Missing | 12.9 |
| **Public care agency leaders’ (N=31) gender identification (%)** | |
| Male | 19.4 |
| Female | 67.7 |
| Missing | 12.9 |
| **Public care agency leaders’ (N=31) level of education (%)** | |
| Bachelor’s degree | 9.7 |
| Master’s degree | 64.5 |
| Doctoral degree | 6.5 |
| Professional degree | 3.2 |
| Missing | 16.1 |
| **Public care agency leaders’ (N=31) racial or ethnic identification (%)** | |
| Hispanic/Latino | 3.2 |
| White | 71.0 |
| African-American/Black | 6.5 |
| Native American/American Indian | 6.5 |
| Missing | 12.9 |
| **Public care agency leaders’ (N=31) role(s) in the PAP (%)** | |
| Contract administrator/manager | 9.7 |
| Government/agency leader | 22.6 |
| Grant manager | 3.2 |
| Partnership broker/liaison | 3.2 |
| Member, unspecified | 12.9 |
| Project/Implementation manager/coordinator | 16.1 |
| Multiple roles | 19.4 |
| Missing | 12.9 |
| **Public care agency leaders’ (N=31) total number of PAPs that they are engaged, including the current PAP (%)** | |
| 1-5 | 74.2 |
| 6-10 | 3.2 |
| 11 or more | 6.5 |
| Not specified | 3.2 |
| Missing | 12.9 |

Note. Of the 48 public care agency leaders, 31 answered the demographics and work experience questionnaire. PAP: Public-academic partnership.
