# Supplementary material for: Purpose Formulation, Coalition Building, and Evidence Use in Public–Academic Partnerships: Web-Based Survey Study
Source: JMIR Hum Factors. 2022 Jan 5;9(1):e29288. doi: 10.2196/29288 (PMC8771345; doi:10.2196/29288)
Supplement: Multimedia Appendix 2 [file humanfactors_v9i1e29288_app2.docx]

| **Academic researchers’ (n=40) age in years (%)** | |
| --- | --- |
| Less than 30 | 0 |
| 30-39 | 15 |
| 40-49 | 37.5 |
| 50-59 | 15.0 |
| 60-69 | 25.0 |
| 70+ | 2.5 |
| Missing | 5.0 |
| **Academic researchers’ (N=40) years of experience in the field (%)** | |
| Fewer than 10 | 10.0 |
| 10-19 | 32.5 |
| 20-29 | 30.0 |
| 30-39 | 17.5 |
| 40 or more | 5.0 |
| Missing | 5.0 |
| **Academic researchers’ (N=40) years in the current organization (%)** | |
| Fewer than 10 | 40.0 |
| 10-19 | 30.0 |
| 20-29 | 22.5 |
| 30-39 | 2.5 |
| Missing | 5.0 |
| **Academic researchers’ (N=40) years involved with current PAP (%)** | |
| Fewer than 10 years | 65.0 |
| 10 or more years | 27.5 |
| Unspecified | 2.5 |
| Missing | 5.0 |
| **Academic researchers’ (N=40) gender identification (%)** | |
| Male | 20.0 |
| Female | 75.0 |
| Missing | 5.0 |
| **Academic researchers’ (N=40) level of education (%)** | |
| Master’s degree | 20.0 |
| Doctoral degree | 65.0 |
| Professional degree | 10.0 |
| Missing | 5.0 |
| **Academic researchers’ (N=40) racial or ethnic identification (%)** | |
| Hispanic/Latino | 2.5 |
| White | 82.5 |
| African-American/Black | 2.5 |
| Asian/Pacific Islander | 5.0 |
| Multi-racial | 2.5 |
| Missing | 5.0 |
| **Academic researchers’ (N=40) role(s) in the PAP (%)** | |
| Director | 17.5 |
| Co-director | 7.5 |
| Project director | 7.5 |
| Principal Investigator/Lead evaluator/University lead | 27.5 |
| Coordination | 5.0 |
| Multiple | 20.0 |
| Other | 7.5 |
| Missing | 7.5 |
| **Academic researchers’ (N=40) total number of PAPs that they are engaged, including the current PAP (%)** | |
| 1-5 | 67.5 |
| 6-10 | 12.5 |
| 11 or more | 5.0 |
| Not specified | 7.5 |
| Missing | 7.5 |

Note. All 40 academic researchers answered the demographics and work experience questionnaire. PAP: Public-academic partnership.
